# Low HDL and high triglycerides predict COVID-19 severity

**DOI:** 10.1038/s41598-021-86747-5

**Published:** 2021-03-30

**Authors:** Lluís Masana, Eudald Correig, Daiana Ibarretxe, Eva Anoro, Juan Antonio Arroyo, Carlos Jericó, Carolina Guerrero, Marcel·la Miret, Silvia Näf, Anna Pardo, Verónica Perea, Rosa Pérez-Bernalte, Núria Plana, Rafael Ramírez-Montesinos, Meritxell Royuela, Cristina Soler, Maria Urquizu-Padilla, Alberto Zamora, Juan Pedro-Botet, the STACOV-XULA research group, Cèlia Rodríguez-Borjabad, Natalia Andreychuk, Ana Malo, Laia Matas, Maria del Señor Cortes-Fernandez, Marta Mauri, Rosa M. Borrallo, Àngels Pedragosa, Pilar Gil-Lluís, Ana Lacal-Martínez, Patricia Barragan-Galló, Glòria Vives-Masdeu, Carmen Arto-Fernández, Omar El Boutrouki, Andrea Vázquez-Escobales, Maria Cristina Antón-Alonso, Sergio Rivero-Santana, Albert Gómez, Sara García, Núria Rial-Lorenzo, Lourdes Ruiz-Ortega, Oriol Alonso-Gisbert, Ana Inés Méndez-Martínez, Hada Iglesias-López, Elisenda Climent, Roberto Güerri, Jade Soldado, Marta Fanlo, Alicia Taboada, Liliana Gutierrez

**Affiliations:** 1 Facultat de Medicina, Universitat Rovira I Virgili, LIPIDCAS, University Hospital Sant Joan, IISPV, CIBERDEM, C/. Sant Llorenç, 21, 43201 Reus, Spain; 2 Statistics Department, Institut Investigació Sanitaria Pere Virgili, Universitat Rovira I Virgili, Reus, Spain; 3 LIPIDCAS, Pius Hospital Valls, Valls, Spain; 4 Lipid Unit, University Hospital Santa Creu I Sant Pau, Barcelona Autonomous University, Barcelona, Spain; 5 Lipid Unit, Hospital Moises Broggi, Consorci Sanitari Integral, Sant Joan Despí, Spain; 6 Internal Medicine Department, Terrasa Hospital, Consorci Sanitari Terrassa, Barcelona, Spain; 7 LIPIDCAS, Endocrinology Department, Hospital Verge de La Cinta, Tortosa, Spain; 8 LIPIDCAS, Endocrinology Department, University Hospital Joan XXIII, IISPV. CIBERDEM, Universitat Rovira I Virgili, Tarragona, Spain; 9 Internal Medicine Department, Hospital Delfos, Barcelona, Spain; 10 Lipid Unit, Hospital Mutua Terrasa, Barcelona, Spain; 11 LIPIDCAS, Hospital del Vendrell, El Vendrell, Spain; 12 LIPIDCAS, Hospital Sant Pau I Santa Tecla, Tarragona, Spain; 13 Lipid Unit, ALTHAIA, Xarxa Assistencial Universitària de Manresa, Barcelona, Spain; 14 Lipid Unit, Hospital Santa Caterina, Girona, Spain; 15 Lipid Unit, University Hospital Vall d’Hebron, Barcelona Autonomous University, Barcelona, Spain; 16 Lipid Unit, Corporació de Salut del Maresme I La Selva, Hospital de Blanes, Blanes, Spain; 17 Lipid Unit, University Hospital del Mar, Barcelona Autonomous University, Barcelona, Spain; 18 Hospital Universitari de Bellvitge, Universitat de Barcelona, Hospitalet de Llobregat, Barcelona, Spain; 19 Hospital de l’ Esperit Sant, Santa Coloma de Gramanet, Barcelona, Spain; 20 Hospital Universitari Arnau de Vilanova, Lleida, Spain

**Keywords:** Dyslipidaemias, Risk factors

## Abstract

Lipids are indispensable in the SARS-CoV-2 infection process. The clinical significance of plasma lipid profile during COVID-19 has not been rigorously evaluated. We aim to ascertain the association of the plasma lipid profile with SARS-CoV-2 infection clinical evolution. Observational cross-sectional study including 1411 hospitalized patients with COVID-19 and an available standard lipid profile prior (n: 1305) or during hospitalization (n: 297). The usefulness of serum total, LDL, non-HDL and HDL cholesterol to predict the COVID-19 prognosis (severe vs mild) was analysed. Patients with severe COVID-19 evolution had lower HDL cholesterol and higher triglyceride levels before the infection. The lipid profile measured during hospitalization also showed that a severe outcome was associated with lower HDL cholesterol levels and higher triglycerides. HDL cholesterol and triglyceride concentrations were correlated with ferritin and D-dimer levels but not with CRP levels. The presence of atherogenic dyslipidaemia during the infection was strongly and independently associated with a worse COVID-19 infection prognosis. The low HDL cholesterol and high triglyceride concentrations measured before or during hospitalization are strong predictors of a severe course of the disease. The lipid profile should be considered as a sensitive marker of inflammation and should be measured in patients with COVID-19.

## Introduction

The COVID-19 pandemic has rapidly spread worldwide, and it appears to be far from being controlled. While the main pathophysiological mechanisms are being revealed, only some of the factors determining whether some patients have an asymptomatic course while others die have been identified. Given the limited treatment options, the identification of risk factors related to severe COVID-19 is of paramount importance to improve the prognosis. Age, sex, background therapies and comorbidities are the main clinical determinants of severity^1–3^. High blood pressure and dysglycaemia are associated with a worse prognosis; however, although dyslipidaemia is one of the main cardiovascular risk factors, the association between baseline lipid levels and the prognosis has been less frequently studied. In this respect, a recent umbrella review of systematic reviews suggested that dyslipidaemia may play a role in the severity of SARS-CoV-2 infection^4^.

Lipids are crucial in the infection process, as they are important structural components of cellular and subcellular organellar membranes. Membrane lipid components participate in the regulation of transmembrane molecular trafficking, including infectious materials such as viruses. Viral internalization requires the attachment of the virus to the host cell membrane, activating an endocytosis mechanism^5^. The membrane lipid composition and particularly the lipid rafts influence this process^6^. Once inside the cell, the virus replicates using the metabolic machinery of the invaded cell^7^. The newly synthesized viral particles exit the cells again by crossing the lipid-rich cell membrane. Based on studies of animal models, intracellular cholesterol may increase cellular SARS-CoV-2 infectivity^8^. Recently, the SREBP-2 C-terminal fragment discovered in blood samples from patients with COVID-19 was identified as an indicator of the severity of the diagnosis and a therapeutic target for preventing the cytokine storm and organ damage^9^. In the host cell, the virus modifies cellular metabolism by altering energy production pathways for its own benefit. Both cellular glucose and lipid metabolism are extensively modified^6^, and a recent metabolomic study of patients infected with SARS-CoV-2 has described a specific lipidomic fingerprint^10^.

In addition to structural and energy supply functions, intracellular lipids also act as intracellular signalling molecules or transcription factors. Viral particles also interfere with these pathways, altering cell physiology and leading to apoptosis and cell death^5^.

The standard clinical approach to assessing alterations in lipid metabolism is based on the measurement of cholesterol in several lipoprotein particles and triglyceride levels. Researchers have not determined how these parameters will improve our understanding of the important role of cellular lipids in viral infections. Infectious diseases are usually associated with low HDL cholesterol (HDL-C) concentrations and sometimes with low LDL cholesterol (LDL-C) concentrations, while triglyceride levels are typically maintained or even increased^6, 11^. Low HDL-C levels have been proposed as a risk biomarker for different infections^12^. Regarding the SARS-CoV-2 infection, low LDL-C, HDL-C and triglyceride (TG) levels have been described to be associated with an increasing infection severity^6^, and a role for these lipids in immune mechanisms has been suggested^13^. Low lipid levels during the infection have been associated with the severity of COVID-19^14^. Meanwhile, low plasma lipid concentrations are regarded as a consequence of the hypermetabolic state and undernutrition in the infected patient; however, many metabolic pathways associated with the immune response and infection itself participate in these alterations^11^. Cytokines, inflammatory mediators, modified lipids and intermediate lipid classes generated during the infection interfere with several steps of lipid metabolism by reducing cholesterol synthesis and absorption, decreasing triglyceride-rich lipoprotein clearance or reducing apolipoprotein (apo) A1 synthesis^15^. Taken together, we hypothesise that an analysis of lipid metabolism should be contemplated as another clinical tool to evaluate the SARS-CoV-2 infection state and prognosis. Thus, the present study aimed to determine the variation in the lipid profile due to COVID-19 and its association with disease severity.

## Results

**Figure 1:**
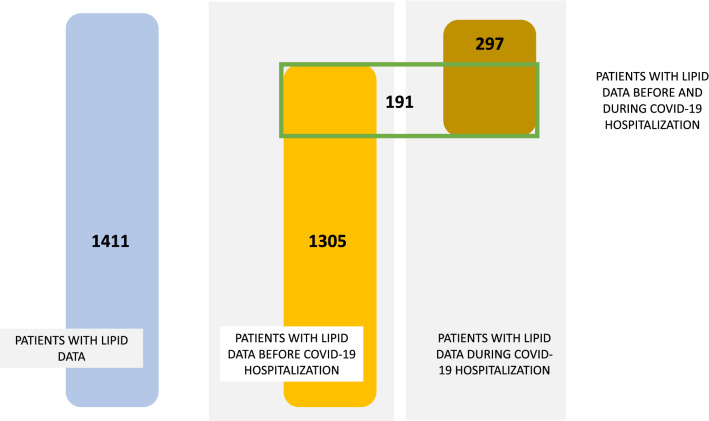
Distribution of patients included in the study according lipid profile availability.

Of the 2159 patients included in the STACOV database, 1305 had an available standard lipid profile (TC, LDL-C, HDL-C, non-HDL-C, TG) before the infection and 297 had a lipid profile that was measured during hospitalization. One hundred ninety-one patients had values for profiles measured at both time points (Fig. 1).

**Figure 2:**
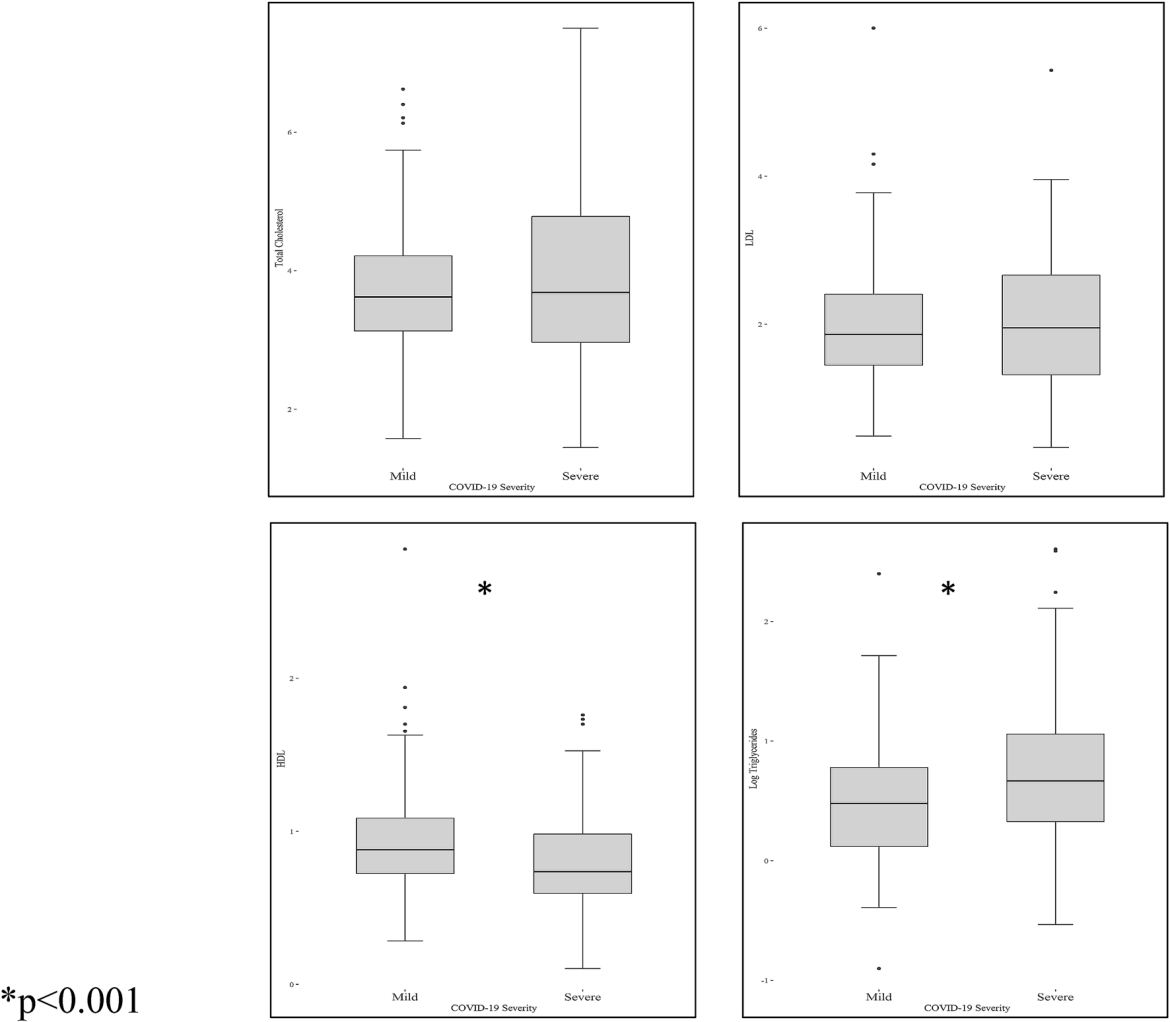
Box plot depicting lipid values obtained during COVID 19 hospitalization sorted according clinical severity.

The demographic and clinical characteristics of the patients included in the study with an available lipid profile before (n = 1305) or during (n = 297) hospitalization sorted by the COVID-19 severity according the aforementioned criteria are shown in supplementary material (Table S1). Regarding the group with pre-infection lipid data, patients with a severe SARS-CoV-2 infection were 6 years older, included a higher proportion of men and as expected, displayed a higher prevalence of comorbidities with significant differences in hypertension, smokers, hyperlipidaemia diagnosis, diabetes, cardiovascular vascular diseases, chronic obstructive pulmonary disease, chronic kidney disease and cancer. While LDL-C levels were similar between patients who developed mild and severe COVID-19, HDL-C concentrations obtained before COVID-19 were lower in patients who developed a severe infection; in contrast, TG levels were significantly higher (Table 1). In the group with a lipid profile that was measured during hospitalization, the demographic and baseline clinical data were better matched between patients who developed a mild or severe disease (supplementary material Table S1). When comparing patients with a mild and severe evolution in this group, again, while LDL-C levels were similar, patients with severe forms of COVID-19 presented significantly lower HDL and higher TG and non-HDL-C concentrations (Table 1 and Fig. 2).

**Table 1:** Lipid profile of patients with data available before or during COVID-19 infection.

Lipid profile available before COVID-19 hospitalization (median [IQR] in mmol/L)*				
	All N = 1305	Mild N = 794	Severe N = 511	P value
Total cholesterol	4.84 [4.14; 5.54]	4,84 [4.17; 5.59]	4.82 [4.07; 5.46]	0.314
HDL cholesterol	1.3[1.09; 1.53]	1.32 [1.09; 1.58]	1.25[1.06; 1.48]	0.007
LDL cholesterol	2.85[2.25; 3.5]	2.87[2.25; 3.5]	2.85 [2.23; 3.42]	0.331
No-HDL cholesterol	3.5 [2.82; 4.2]	3.5 [2.8; 4.25]	3.52 [2.85; 4.1]	0.845
Triglycerides	1.37 [0.99; 1.87]	1.31 [0.95; 1.78]	1.44 [1.06; 1.99]	< 0.001

Lipid profile available during COVID-19 hospitalization				
	All N = 297	Mild N = 149	Severe N = 148	P value
Total cholesterol	3.65 [3.03; 4.35]	3.63 [3.13; 163]	3.68 [2.97; 4.79]	0.528
HDL cholesterol	0.8 [0.64; 1.04]	0.88 [0.72; 1.08]	0.73 [0.59; 0.98]	< 0.001
LDL cholesterol	1.90 [1.39; 2.53]	1.86 [1.45; 2.40]	1.95 [1.32; 2.66]	0.757
No-HDL cholesterol	2.82 [2.15; 3.41]	2.79 [2.12; 3.26]	2.97 [2.19; 3.78]	0.033
Triglycerides	1.76 [1.22; 2.47]	1.61 [1.13; 2.18]	1.94 [1.39; 2.88]	< 0.001

*Median [interquartile range]. To convert mmol/L to mg/dl: cholesterol values × 38.6; triglyceride values × 88.4.

Total cholesterol (TC), LDL-C, non-HDL-C and HDL-C concentrations obtained before COVID-19 were significantly higher than the levels measured during hospitalization. In contrast, higher TG levels were measured during the infection (Table 1).

When examining the lipid profile obtained during hospitalization, the median HDL concentrations were significantly reduced by 16% in patients with a severe disease while the TG concentrations were 20% higher (Table 1).

**Figure 3:**
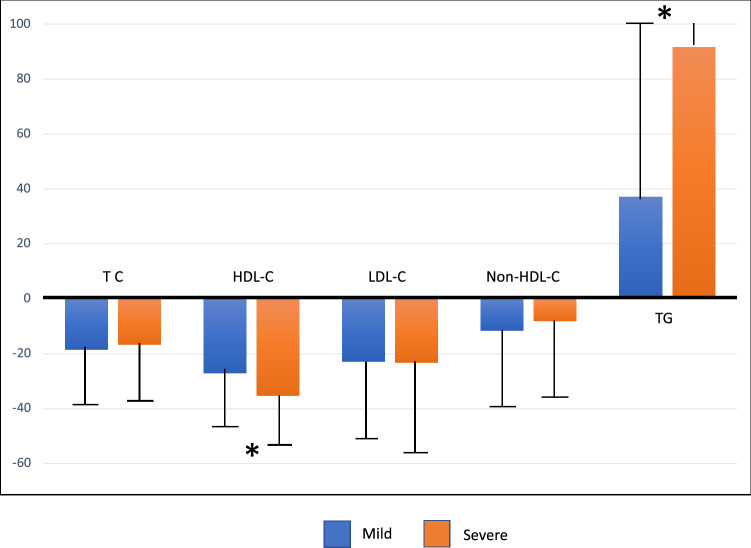
Percentage variation of mean (SD) lipid values in the group with data before and during hospitalization sorted by COVID-19 severity. *TC* total cholesterol, *TG* triglycerides; *p < 0.001.

These differences were confirmed in the group in which a lipid profile was available before and during COVID-19 (n = 191), showing statistically significant differences in all lipid parameters (Fig. 3). Interestingly, in this specific group that allowed a direct comparison, the percent variation of mean values in HDL-C and triglyceride levels was significantly higher for those patients who developed serious COVID-19 (Fig. 3).

**Figure 4:**
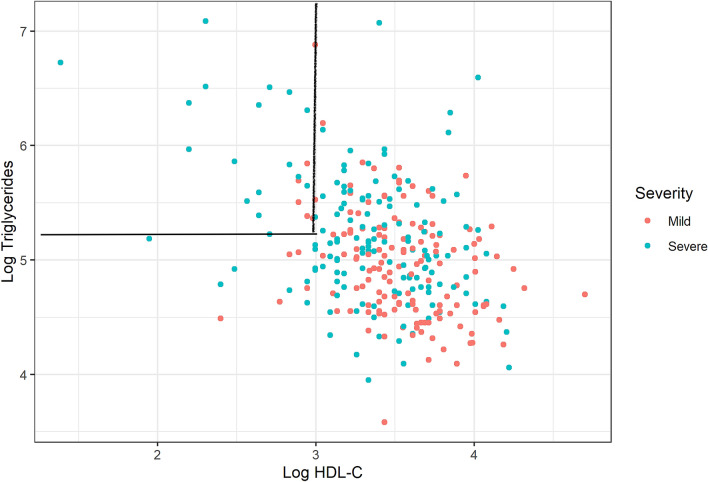
Distribution of patients according HDL-C and TG concentrations and COVID-19 severity. The upper left square shows that only severe patients were in the low HDL-C, high triglyceride area.

As shown in Fig. 4, patients who developed severe symptoms tended to be grouped in the low HDL-C and high TG area.

**Figure 5:**
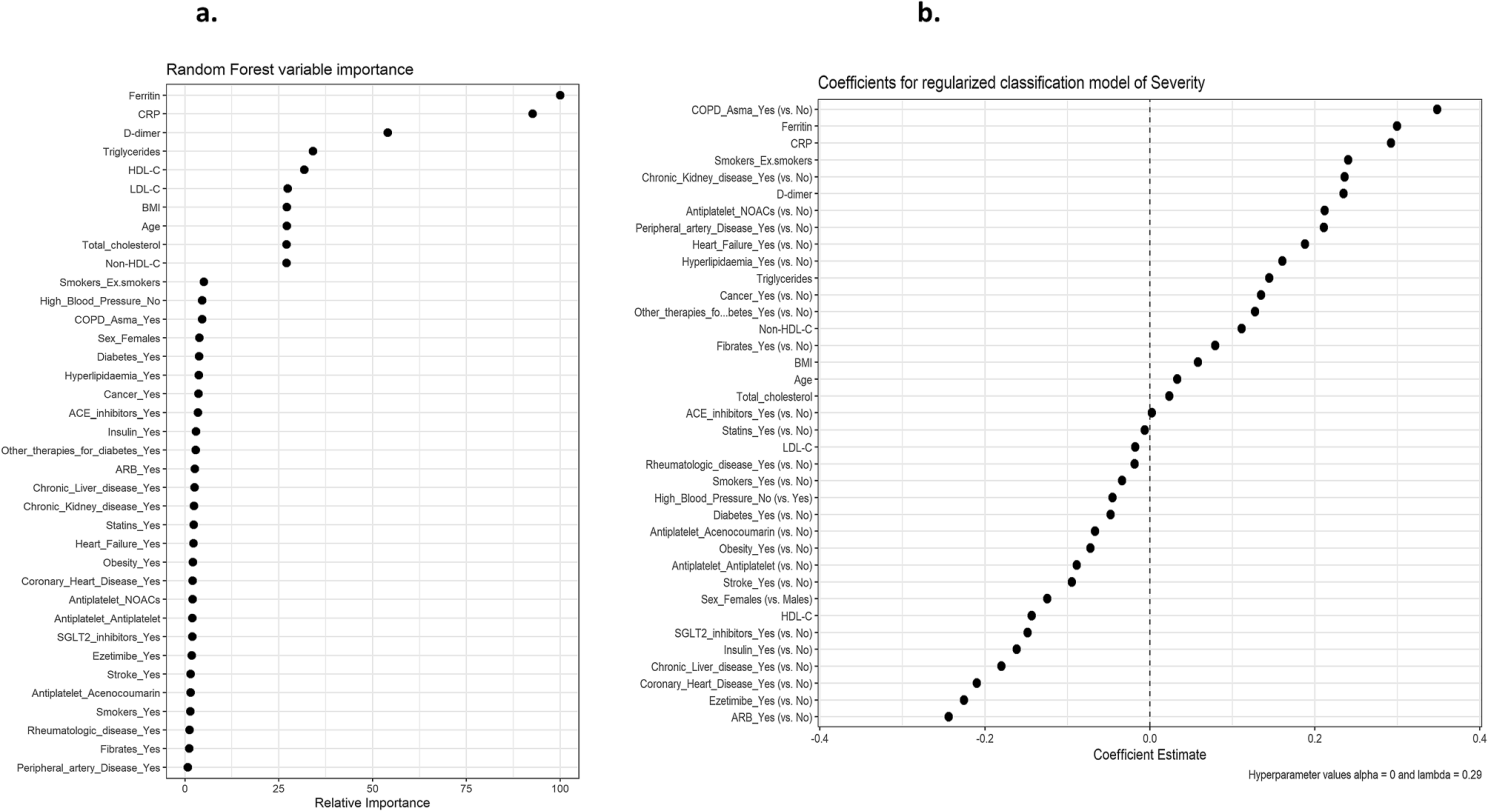
(**a**) Plot depicting the out of bag importance of each analysed variable on COVID-19 random forest model. P values of the variables of interest according a variable permutation analysis: Ferritin: 0.017; CRP: 0.009; D-Dimer: < 0.001; triglycerides: 0.006; HDL-C: 0.019; LDL-C: 0.026; Body mass index: 0.008; age: < 0.001; total cholesterol:  < 0.001; non-HDL-C: < 0.001. (**b**) Coefficients for regularized classification model of severity applicable to (**a**) data indicating the direction of the effect.

Triglyceride concentrations showed a weak but significant direct correlation with ferritin levels (R: 0.25; p < 0.05), while HDL-C levels correlated inversely with ferritin (R: − 0.28; p < 0.01) and D-dimer (R: − 0.016; p < 0.05) concentrations. CRP levels were not significantly correlated with any lipid parameter. We also performed a multivariate analysis as outlined in the statistical methods section to assess the effectiveness of lipid parameters as COVID-19 biomarkers. The highest performing model was a random forest model, with an accuracy of 76% for the test set and an AUC of 0.84 (95% CI 0.79–0.88). The most important variables in this model were Ferritin, CRP and D-dimer, followed closely by the lipid profile, specifically TG, HDL-cholesterol and LDL-cholesterol levels, which showed the same importance level as age and body mass index (Fig. 5a). In Fig. 5b we show the sign of the impact of each variable. As expected, high TG and low HDL-C levels were associated with a worse prognosis.

## Discussion

As shown in the present study, plasma lipid concentrations should assist with the clinical management of COVID-19. Including lipid profile alterations as one of the poor prognostic criteria could help the decision-making process driving to an earlier treatment intensification. Our data are sufficiently robust for us to conclude that low HDL-C levels and high triglyceride levels during hospitalization should be considered high risk markers for COVID-19. On the other hand, in patients with an available pre-infection lipid profile, low HDL cholesterol levels and high triglyceride levels are also associated with a worse prognosis of COVID 19.

Surprisingly, only 297 of the 2159 patients (13.7%) included in the database had a lipid profile that was determined during hospitalization, which was lower than other inflammatory markers, such as CRP or ferritin. Lipids are usually associated with the nutritional status of patients, but not as markers of infection or inflammation. Our data support the relevance of lipids, even the standard lipid profile, as markers of the global inflammatory status and helpful risk markers.

Several studies have described rapid changes in the lipid profile in response to COVID-19 and the progression of the disease^4^. Therefore, a major gap may exist between the presence of dyslipidaemia prior to viral infection and the time at which lipid disturbances were detected at the time of hospitalization. In the present study, infected patients presented lower TC, LDL-C, HDL-C and non-HDL-C levels compared to the levels measured before the disease; however, TG levels were significantly higher (Table 1 and Fig. 3). Several explanations for these variations have been proposed. A hypercatabolic status and the usual undernourishment during acute infections must play a role; however, the paradoxical behaviour of triglyceride concentrations requires additional explanation. Of interest, patients with a severe evolution presented significantly higher triglyceride and lower HDL-C levels, suggesting that factors associated with COVID 19 evolution influence these lipid parameters. In the group of patients with a lipid profile that was determined before the infection, patients with a worse clinical outcome were older and had more comorbidities, which might influence the differences in the levels of both lipids^16, 17^ and outcomes; however, in the group where lipids were measured at admission, no age-related differences were observed, and the comorbidity burden was balanced (supplementary material, table S1). A possible effect of background therapies or COVID-19 treatments should also be considered. While no important differences in baseline therapies, including statin therapy^18^, was observed, patients with a worse COVID-19 evolution received more special therapies. More patients with severe COVID-19 were on corticosteroid therapy, which might be responsible for the high triglyceride levels; however, the lack of an effect on LDL and HDL cholesterol levels, the fact that the lipid profile is usually determined at admission and the result from the multivariate analysis suggesting the independent role of lipid parameters in determining the COVID-19 prognosis indicates that the pharmacological effect is not the only explanation for our observations^19, 20^. The combination of low HDL levels and high triglyceride levels, which is referred to as atherogenic dyslipidaemia, is associated with qualitative changes in LDL particles that lead to an increase in the levels of small and dense LDL particles. This pattern is driven by insulin resistance and usually associated with diabetes and obesity. However, in our patients, the body mass index and the prevalence rates of diabetes and obesity were similar between groups with a mild or severe COVID-19 evolution.

Strong scientific evidence supports the hypothesis that both inflammatory states and infectious diseases are associated with striking changes in lipid metabolism^21^. The general pattern is exactly as observed in our study: low HDL-C levels and normal or even high levels of triglycerides for the clinical context. LDL-C levels may be maintained or also reduced but are generally associated with an increased presence of smaller LDL particles. Although insulin resistance plays an important role in these lipid abnormalities, the pathophysiology of these modifications is more complex. A key enzyme participating in these alterations is lipoprotein lipase^15^ (LPL). In the presence of inflammation, several mediators interfere with glucose and lipid metabolism^22^. Several cytokines and inflammatory mediators that are overexpressed during COVID-19 may interact directly with LPL or its regulatory proteins, such as apo CII^23^. Among these proteins, several products delivered by activated macrophages, such as tumour necrosis factor (TNF), interleukin (IL)-1, IL-11, and Interferon-γ^24, 25^, as well as products derived from bacteria, such as lipopolysaccharide (LPS) associated with septicaemia, and modified lipoproteins such as oxidized LDL or lysophosphatidylcholine have been shown to inhibit LPL activity^15, 21, 26^.

Lower LPL activity results in a decreased conversion of triglyceride-rich lipoproteins (TRL) to LDL, leading to high TG and low HDL levels. Moreover, altered activity of the cholesteryl ester transfer protein (CETP) leading to smaller LDL particles and lower HDL concentrations has also been described and associated with a poor infection prognosis^27, 28^. Although some of these pathways may partially explain the low HDL levels, other factors, including the inhibition of apo A1 synthesis or increased HDL clearance due to the uptake of inflammatory mediators such as serum amyloid A (SAA) by HDL, must be taken into consideration^11^.

Recent plasma lipidomic data from patients infected with SARS-CoV-2 have revealed substantial modifications of lipid families during the infection. Plasma monosialodihexosyl ganglioside concentrations were negatively correlated with CD4 + T cells and positively correlated with disease severity^10^. Interestingly, clinical lipid parameters were differentially correlated with lipid families. In general, cholesterol levels in both apo B and apo A1 lipoproteins tend to display direct correlations with plasma phosphatidylcholine levels and extracellular vesicle sphingomyelin levels, while triglyceride concentrations were more strongly associated with phosphatidylethanolamine levels^10^. Although the clinical implications of these observations remain to be completely elucidated, they provide evidence for a relationship between standard lipids measured at the clinical level and lipid derangements throughout COVID-19.

Lipoprotein modifications observed during COVID-19, as in other infectious situations, should not be considered as only a marker of severity or a surrogate marker of inflammation, as they may have a pathogenic role. Triglyceride-rich lipoproteins have been associated with innate immunity^26^ due to their capacity to bind toxins. Furthermore, low HDL cholesterol levels may cause dysfunction in innate immunity, a first-line defence mechanism against COVID-19^29^. HDL is an inflammatory mediator that buffers toxic molecules by absorbing them and transporting them to the liver for clearance^11^. Therefore, the structural and functional changes observed during COVID-19 might influence the disease evolution.

The present study has some limitations. First, it was a retrospective study, precluding any analysis of the causal relationship in our results. Second, the lipid concentrations measured before the infection were obtained at any moment during the previous year; therefore, we were unable to ascertain the proximity of these concentrations to the clinical event. Similarly, we were unable to precisely determine when the lipid profile was measured during hospitalization for COVID-19, although it is usually included in the first standard blood test performed after admission; therefore, we were only able to deduce that the lipid profile was measured within the first 48 h. Finally, only 191 patients had both measurements before and during the infection, limiting our ability to obtain robust conclusions about the effect of lipid variations during the infection.

Standard lipid concentrations are not only a marker of nutritional status but also a biomarker of infection and the degree of inflammation that may play a pathophysiological role. A high triglyceride level associated with a low HDL-C concentration must be considered as a marker of a poor prognosis. Our data strongly support the need for lipid profile measurements during the course of COVID-19, since the lipid profile appears to be a potent risk biomarker.

## Methods

### Study design

This study employed a observational cross-sectional design. Data from patients included in the STACOV cohort (Cohort registration code NCT04407273-STACOV) with an available lipid profile were included in the analysis. Clinical characteristics of the STACOV cohort have been described previously^18^. Briefly, members of the Lipids and Arteriosclerosis Units Net (XULA) of Catalonia (Spain) retrieved clinical data collected from patients admitted to their hospitals because of a SARS-CoV-2 infection up to May 31, 2020. Consecutive patients aged at least 18 years who were hospitalized for at least 24 h were eligible. Only data from patients with a definite COVID-19 diagnosis obtained by reverse transcription-polymerase chain reaction (PCR) whose infections were acquired in the community were included. A total of 2159 patients were included in the final database.

### Data acquisition and confidentiality

All collected data were anonymized, preventing reidentification. All procedures were conducted in accordance with the legal provisions of the protection of personal data in Spain and according the European Union Regulations (EU) 2016/6799 on the physical protection of the treatment of personal data. The study was compliant with the Declaration of Helsinki. It was approved by the Ethics Committee of the University Research Institute “Pere Virgili” (Reus), including the exemption of the requirement for informed consent.

### Eligibility criteria

Only those patients from the STACOV database with an available lipid profile (TC, LDL-C, HDL-C, non-HDL-C, and TG levels) that was measured within the previous year or during hospitalization were included to study the effect of circulating lipid and lipoprotein levels on patients who were admitted to hospital for COVID-19.

### Main objective

The main objective of the study was to assess the relationship between standard serum lipid concentrations measured before or during hospitalization for COVID-19 with the infection evolution. Infection severity was categorized as mild (patients without oxygen therapy or oxygen provided only by a mask or nasal prongs) or severe (patients requiring ventilation, high flow oxygen, mechanical ventilation, or special therapy for organ function support, and patients who died).

Lipid variations associated with COVID-19 and their correlations to clinical variables and effects on clinical severity were also analysed.

### Statistical analyses

Continuous variables were tested for normality using the Shapiro–Wilk test. Data are presented as medians and 25th and 75th percentiles for continuous variables with a non-normal distribution or as the means and standard deviations (SDs) for variables with a normal distribution. Unless indicated otherwise, categorical variables are reported as percentages. Differences between groups were analysed using the non-parametric Mann–Whitney test or Student’s parametric t test for continuous variables and the chi-square test or Fisher’s exact test for categorical variables. All continuous variables were standardized and normalized when necessary.

Regarding the multivariate test, we constructed a series of predictive models to assess the relationship between clinical and lipid variables and the COVID-19 prognosis (mild vs. severe). The methodology is as follows: first, we divided our dataset into a training set, consisting of 80% of the observations (patients), in which models were constructed, and a test set, with the other 20%, where we evaluated the model performance. Two types of models were used: logistic regression models regularized via an elastic net and random forest models. For each scenario, we trained several versions of each type of model and adjusted their parameters via fivefold cross-validation and chose the model with better performance. This model was then evaluated using the test set, and its performance is the one that we report.

The system under study proved to be rather complex, especially due to the non-linearity of the relationships between predictor and predicted variables and also because of the relations between predictor variables. It's for this reason that we resorted to Random Forests models, which are able to capture non-linearity as well as accommodate relations between predictor variables and are much better suited for these complex scenarios^30^. Furthermore, they allow us to assess the relative importance and p-value of each variable against all others by determining the out-of-bag accuracy and importance before and after variable permutation^31^. However, these models do not provide information as to the protective or harmful effect of each variable on the prognosis of patients with COVID-19. Since this assessment is clearly a very important measure in our clinical setting, we also computed the regularized logistic regression analyses, through which we evaluated the direction of the effect based on the sign of the coefficient. Since this effect is the only parameter in which we are interested and variable importance will be inferred from the non-linear models, confidence intervals and p-values were not reported. As we shall see, both models were slightly different in terms of variable selection, but sufficiently similar to be able to combine them to draw meaningful conclusions.

Statistical analyses were performed using the R software package version 3.5. (R Core Team. R: A language and environment for statistical computing. R Foundation for Statistical Computing. 2020. Vienna, Austria. URL: https://www.R-project.org/).

The dataset supporting the conclusions of this article is available in https://osf.io/mwc8n/?view_only=82bb2153fbb04aad890a3fb266fc7ba4 previous requirement to authors.

## Supplementary Information

Supplementary Information
