# Supplementary material for: Low HDL and high triglycerides predict COVID-19 severity
Source: Sci Rep. 2021 Mar 30;11:7217. doi: 10.1038/s41598-021-86747-5 (PMC8010012; doi:10.1038/s41598-021-86747-5)
Supplement: Supplementary file 1 — Supplementary Information [file 41598_2021_86747_MOESM1_ESM.pdf]

LOW HDL AND HIGH TRIGLYCERIDES PREDICT COVID-19 SEVERITY

Lluís Masana*[1], Eudald Correig[2], Daiana Ibarretxe[1], Eva Anoro[3], Juan Antonio Arroyo[4], Carlos Jericó[5], Carolina Guerrero[6], Marcel·la Miret[7], Silvia Näf[8], Anna Pardo[9], Verónica Perea[10], Rosa Pérez-Bernalte[11], Núria Plana[1], Rafael Ramírez-Montesinos[12], Meritxell Royuela[13], Cristina Soler[14], Maria Urquizu-Padilla[15], Alberto Zamora[16], and Juan Pedro-Botet[17] on behalf of the STACOV-XULA research group

1. Universitat Rovira i Virgili, LIPIDCAS, University Hospital Sant Joan IISPV, CIBERDEM, Reus, Spain.
2. Universitat Rovira i Virgili. Statistics Department, Institut Investigació Sanitaria Pere Virgili, Reus, Spain.
3. LIPIDCAS, Pius Hospital Valls, Valls, Spain.
4. Lipid Unit, University Hospital Santa Creu i Sant Pau, Barcelona Autonomous University, Barcelona, Spain.
5. Lipid Unit, Hospital Moises Broggi. Consorci Sanitari Integral. Sant Joan Despí, Spain.
6. Internal Medicine Department. Terrasa Hospital. Consorci Sanitari Terrassa, Spain.
7. LIPIDCAS, Endocrinology Department, Hospital Verge de la Cinta, Tortosa, Spain.
8. LIPIDCAS, Endocrinology Department, University Hospital Joan XXIII, IISPV. CIBERDEM. Universitat Rovira i Virgili. Tarragona, Spain
9. Internal Medicine Department, Hospital Delfos, Barcelona, Spain.
10. Lipid Unit, Hospital Mutua Terrasa, Barcelona, Spain.
11. LIPIDCAS, Hospital del Vendrell, El Vendrell, Spain.

12. LIPIDCAS, Hospital Sant Pau i Santa Tecla, Tarragona, Spain
13. Lipid Unit, ALTHAIA, Xarxa Assistencial Universitària de Manresa, Spain.
14. Lipid Unit, Hospital Santa Caterina, Girona, Spain.
15. Lipid Unit, University Hospital Vall d'Hebron, Barcelona Autonomous University, Barcelona, Spain.
16. Lipid Unit, Corporació de Salut del Maresme i la Selva, Hospital de Blanes, Spain.
17. Lipid Unit, University Hospital del Mar, Barcelona Autonomous University, Barcelona, Spain.

Correspondence to:

Lluís Masana

Facultat de Medicina URV

c/. Sant Llorenç, 21

43201- Reus (Spain

Telephone: +34 977759371

e-mail: luis.masana@urv.cat

TABLE S1.

Baseline clinical data of hospitalized COVID-19 patients sorted by infection severity.

Clinical data at baseline and during hospitalization for COVID-19 infection in patients with lipid profile available before or during infection, sorted by severity of COVID-19 evolution.

	Patients with lipid values available BEFORE hospitalization				Patients with lipid values available DURING hospitalization			
	**All N(%)**	**Mild N(%)**	**Severe N(%)**	**P value**	**All N(%)**	**Mild N(%)**	**Severe N(%)**	**P value**
**NUMBER**	1305	794	511		297	149	148	
**DEMOGRAPHIC AND BASELINE CLINICAL DATA**								
**Age (SD)**	67.4 (14.7)	65.1 (15.0)	71.1 (13.2)	<0.001	64.2 (14.6)	63.5 (15.7)	64.9 (13.5)	0.394
**Sex , women**	567 (43.4)	374 (47.1)	193 (37.8)	<0.001	100 (33.7)	60 (40.3)	40 (27.0)	0.022
**Tobacco**	64 (4.90)	34 (4.28)	30 (5.87)	<0.001	20 (6.73)	11 (7.38)	9 (6.08)	0.054

**High Blood Pressure**	719 (55.1)	399 (50.3)	320 (62.6)	<0.001	149 (50.2)	72 (48.3)	77 (52.0)	0.601
**Hyperlipidaemia**	601 (46.1)	327 (41.2)	274 (53.6)	<0.001	114 (38.4)	50 (33.6)	64 (43.2)	0.110
**Diabetes**	368 (28.2)	196 (24.7)	172 (33.7)	0.001	86 (29.0)	43 (28.9)	43 (29.1)	1.000
**Obesity**	469 (35.9)	287 (36.1)	182 (35.6)	0.892	78 (26.3)	38 (25.5)	40 (27.0)	0.868
**Body Mass Index (SD)**	31.8 (3.6)	33.1 (4.2)	29.7 (5.99)	0.305	28.2 (3.86)	28.0 (3.78)	28.5 (3.93)	0.233
**Coronary Heart Disease**	145 (11.1)	80 (10.1)	65 (12.7)	0.163	22 (7.41)	14 (9.40)	8 (5.41)	0.275
**Stroke**	82 (6.28)	41 (5.16)	41 (8.02%)	0.050	19 (6.40)	11 (7.38)	8 (5.41)	0.646
**Peripheral Artery Disease**	70 (5.36)	34 (4.28)	36 (7.05)	0.042	14 (4.71)	5 (3.36)	9 (6.08)	0.404
**Heart Failure**	124 (9.50)	67 (8.44)	57 (11.2)	0.124	17 (5.72)	7 (4.70)	10 (6.76)	0.607
**Chronic Obstructive Pulmonary disease**	245 (18.8)	125 (15.7)	120 (23.5)	0.001	34 (11.4)	10 (6.71)	24 (16.2)	0.017
**Chronic Liver Disease**	30 (2.30)	19 (2.39)	11 (2.15)	0.925	9 (3.03)	5 (3.36)	4 (2.70)	1.000

**Chronic Kidney Disease**	152 (11.6)	72 (9.07)	80 (15.7)	<0.001	19 (6.40)	8 (5.37)	11 (7.43)	0.625
**Rheumatic Disease**	66 (5.06)	40 (5.04)	26 (5.09)	1.000	9 (3.03)	5 (3.36)	4 (2.70)	1.000
**Cancer**	147 (11.3)	78 (9.82)	69 (13.5)	0.050	31 (10.4)	13 (8.72)	18 (12.2)	0.436
**BACKGROUND PHARMACOLOGICAL THERAPY**								
**Statins**	445 (34.1)	249 (31.4)	196 (38.4)	0.011	80 (26.9)	39 (26.2)	41 (27.7)	0.868
**Ezetimibe**	33 (2.53)	20 (2.52)	13 (2.54)	1.000	11 (3.70)	6 (4.03)	5 (3.38)	1.000
**Fibrate**	43 (3.30)	18 (2.27)	25 (4.89)	0.015	15 (5.05)	6 (4.03)	9 (6.08)	0.587
**Angiotensin Converting Enzyme Inhibitors**	311 (23.8)	178 (22.4)	133 (26.0)	0.154	71 (23.9)	34 (22.8)	37 (25.0)	0.761
**Angiotensin Receptor Blockers**	205 (15.7)	118 (14.9)	87 (17.0)	0.332	39 (13.1)	23 (15.4)	16 (10.8)	0.313
**Insulin**	121 (9.27)	61 (7.68)	60 (11.7)	0.018	27 (9.09)	15 (10.1)	12 (8.11)	0.700

**Sodium Glucose co-Transporter Inhibitors**	29 (2.22)	13 (1.64)	16 (3.13)	0.111	10 (3.37)	5 (3.36)	5 (3.38)	1.000
**Glucagon Like Peptide 1 receptor agonists**	20 (1.53)	13 (1.64)	7 (1.37)	0.878	3 (1.01)	2 (1.34)	1 (0.68)	1.000
**Other Diabetes Therapies**	275 (21.1)	149 (18.8)	126 (24.7)	0.013	59 (19.9)	27 (18.1)	32 (21.6)	0.541
**Antiplatelet**	254 (19.5)	130 (16.4)	124 (24.3)	0.001	47 (15.8)	26 (17.4)	21 (14.2)	0.769
**New Oral Anticoagulants**	60 (4.60)	35 (4.41)	25 (4.89)	0.001	15 (5.05)	6 (4.03)	9 (6.08)	0.769
**Acenocoumarol**	79 (6.05)	42 (5.29)	37 (7.24)	0.001	16 (5.39)	8 (5.37)	8 (5.41)	0.769
**HEMATOLOGICAL AND BIOCHEMICAL BIOMARKERS DURING HOSPITALIZATION**								
**Total Leucocytes x10^9 /L[IQR]**	6.520 [4.000;8.940]	6.035 [3.800;7710]	7.980 [4.500;11.322]	<0.001	6.900 [4.280;9920]	5.590 [3.420;7750]	8.590 [5.642;12.283]	<0.001
**Lymphocytes x10^9 /L [IQR]**	0.680 [0.310;0.950]	0.800 [0.420;1.050]	0.520 [0.295;0.753]	<0.001	0.700 [0.420;0.999]	0.800 [0.500;1.121]	0.600 [0.400;0.900]	0.001

**Ferritin,µg/L[IQR]**	1124 (1083)	805 (695)	1620 (1357)	<0.001	909 [452;1728]	568 [272;1067]	1348 [854;2000]	<0.001
**C-Reactive Protein,µg/ml[IQR]**	238 [93.9;1050]	168 [69.1;617]	353 [164;1710]	<0.001	201 [93.5;380]	120 [57.1;226]	295 [174;475]	<0.001
**D-Dimer,µg/L[IQR]**	1080 [530;2633]	808 [448;1630]	2198 [804;4772]	<0.001	1540 [710;3930]	1050 [570;2330]	2587 [1088;5932]	<0.001
**PaO2, kPa [IQR]**	9.06 [7.86;10.4]	9.48 [8.66;10.7]	8 [6.93;9.46]	<0.001	9.33 [7.42;10.8]	10.25 [8.93;11.4]	7.86 [6.93;9.62]	<0.001
**Pa02/Fi02 [IQR]**	38.1 [28;45]	42.8 [37.5;47.6]	25.3[14.6;35.3]	<0.001	33.4 [19.5;43]	42.6[36.9;45]	19.7 [13.2;27.3]	<0.001
**COVID-19 THERAPY**								
**Chloroquine**	1158 (88.7)	724 (91.2)	434 (84.9)	0.001	267 (89.9)	130 (87.2)	137 (92.6)	0.184
**Antibiotics**	1154 (88.4)	688 (86.6)	466 (91.2)	0.016	270 (90.9)	130 (87.2)	140 (94.6)	0.045
**Anti-retroviral**	811 (62.1)	463 (58.3)	348 (68.1)	<0.001	185 (62.3)	81 (54.4)	104 (70.3)	0.007
**Corticosteroids**	409 (31.3)	153 (19.3)	256 (50.1)	<0.001	116 (39.1)	30 (20.1)	86 (58.1)	<0.001
**Immunomodulators**	242 (18.5)	69 (8.69)	173 (33.9)	<0.001	59 (19.9)	7 (4.70)	52 (35.1)	<0.001

**Immunoglobulins**	20 (1.53)	5 (0.63)	15 (2.94)	0.002	7 (2.36)	2 (1.34)	5 (3.38)	0.282
**Anticoagulation**	149 (11.4)	57 (7.18)	92 (18.0)	<0.001	32 (10.8)	10 (6.71)	22 (14.9)	0.001
**COVID-19 CLINICAL OUTCOMES**								
**Intensive Care unit**	212 (16.2)	5 (0.63)	207 (40.5)	<0.001	01 (34.0)	3 (2.01)	98 (66.2)	<0.001
**Bilateral alteration in Chest X Ray**	981 (75.2)	527 (66.4)	454 (88.8)	<0.001	235 (79.1)	102 (68.5)	133 (89.9)	<0.001
**Shock**	95 (7.28)	3 (0.38)	92 (18.0)	<0.001	38 (12.8)	1 (0.67)	37 (25.0)	<0.001
**Acute Respiratory Distress Syndrome**	403 (30.9)	0 (0.00)	403 (78.9)	<0.001	132 (44.4)	0 (0.00)	132 (89.2)	<0.001
**Intravascular disseminated Coagulation**	21 (1.61)	0 (0.00)	21 (4.11)	<0.001	11 (3.70)	0 (0.00)	11 (7.43)	0.002
**Renal Function Alterations**	222 (17.0)	64 (8.06)	158 (30.9)	<0.001	62 (20.9)	13 (8.72)	49 (33.1)	<0.001

**Liver Enzymes Alterations**	41 (3.14)	6 (0.76)	35 (6.85)	<0.001	4 (4.71)	4 (2.68)	10 (6.76)	0.197
**High-Flow Mechanical Ventilation**	267 (20.5)	0 (0.00)	267 (52.2)	<0.001	80 (26.9)	0(0.00)	80 (54.0)	<0.001
**Invasive Mechanical Ventilation.**	174 (13.3)	0 (0.00)	174 (34.1)	<0.001	84 (28.3)	0 (0.00)	84 (56.8)	<0.001
**Death**	218 (16.7)	0 (0.00)	218 (42.7)	<0.001	44 (14.8%)	0 (0.00%)	44 (29.7%)	<0.001
